# The Bifidogenic Effect of 2’Fucosyllactose Is Driven by Age-Specific *Bifidobacterium* Species, Demonstrating Age as an Important Factor for Gut Microbiome Targeted Precision Medicine

**DOI:** 10.3390/nu17010151

**Published:** 2024-12-31

**Authors:** Jenni Firrman, Stef Deyaert, Karley K. Mahalak, LinShu Liu, Aurélien Baudot, Marie Joossens, Jonas Poppe, Simon J. S. Cameron, Pieter Van den Abbeele

**Affiliations:** 1 United States Department of Agriculture, Agricultural Research Service, Eastern Regional Research Center, Dairy and Functional Foods Research Unit, 600 East Mermaid Lane, Wyndmoor, PA 19462, USA; Jenni.Firrman@usda.gov (J.F.);; 2 Cryptobiotix, Technologiepark-Zwijnaarde 82, 9052 Gent, Belgium; 3 Laboratory of Microbiology, Department of Biochemistry and Microbiology (WE10), Ghent University, 9000 Ghent, Belgium; 4 School of Biological Sciences and Institute for Global Food Security, Queen’s University Belfast, 19 Chlorine Gardens, Belfast BT9 5DL, Northern Ireland, UK

**Keywords:** 2’-fucosyllactose, *Bifidobacterium*, prebiotic, systemic intestinal fermentation research (SIFR^®^), gut microbiota, precision medicine, senescence, LA-REIMS, metagenomics

## Abstract

Background: The human gut microbiota develops in concordance with its host over a lifetime, resulting in age-related shifts in community structure and metabolic function. Little is known about whether these changes impact the community’s response to microbiome-targeted therapeutics. Providing critical information on this subject, faecal microbiomes of subjects from six age groups, spanning from infancy to 70-year-old adults (n = six per age group) were harvested. The responses of these divergent communities to treatment with the human milk oligosaccharide 2’-fucosyllactose (2’FL), fructo-oligosaccharides (FOS), and lactose was investigated using the *Ex vivo* SIFR^®^ technology that employs bioreactor fermentation and is validated to be predictive of clinical findings. Additionally, it was evaluated whether combining faecal microbiomes of a given age group into a single pooled microbiome produced similar results as the individual microbiomes. Results: First, marked age-dependent changes in community structure were identified. *Bifidobacterium* levels strongly declined as age increased, and *Bifidobacterium* species composition was age-dependent: *B. longum*, *B. catenulatum/pseudocatenulatum,* and *B. adolescentis* were most prevalent for breastfed infants, toddlers/children, and adults, respectively. Metabolomic analyses (LA-REIMS) demonstrated that these age-dependent differences particularly impacted treatment effects of 2’FL (more than FOS/lactose). Further analysis revealed that while 2’FL enhanced production of short-chain fatty acids (SCFAs) and exerted potent bifidogenic effects, regardless of age, the specific *Bifidobacterium* species enhanced by 2’FL, as well as subsequent cross-feeding interactions, were highly age-dependent. Furthermore, single-pooled microbiomes produced results that were indicative of the average treatment response for each age group. Nevertheless, pooled microbiomes had an artificially high diversity, thus overestimating treatment responses (especially for infants), did not recapitulate interindividual variation, and disallowed for the correlative analysis required to unravel mechanistic actions. Conclusions: Age is an important factor in shaping the gut microbiome, with the dominant taxa and their metabolites changing over a lifetime. This divergence affects the response of the microbiota to therapeutics, demonstrated in this study using 2’FL. These results evidence the importance of screening across multiple age groups separately to provide granularity of how therapeutics impact the microbiome and, consequently, human health.

## 1. Background

There is a well-established link between the gut microbiota, nutrition, and human health [1]. This association is used advantageously by developers of prebiotics, probiotics, or therapeutics that target the gut microbiome to maintain or restore human health, or potentially prevent or ameliorate disease symptoms [2]. However, understanding and predicting the benefits of delivered therapeutics is often complicated by the individualistic nature of the gut microbiota, which uniquely coevolves with its host in response to a number of factors, i.e., the environment, genetic background, diet, and medication usage [3,4,5]. As these factors change for the host over the course of a lifetime so does the gut microbiota, and although the gut microbes themselves do not age, there is a documented age-related shift in this community’s structure and function that occurs [6,7,8].

Beginning after birth, the infant gut microbiome has low diversity that increases with age, and a high prevalence of *Bifidobacterium* [9]. By 3 years of age, the gut microbiota is estimated to share 40–60% similarly with an adult, showing that the community evolves towards an adult-like state during the first years of life [10,11,12]. As a person ages, there are again specific changes that occur such as decreases in abundance of *Bifidobacterium* and *Faecalibacterium prausnitzii*, in contrast to increased levels of *Escherichia coli* and Bacteroidota as reported for elderly adults [9,10,11,12]. Functionally, for adults, it has been previously reported that levels of short-chain fatty acids (SCFAs) are reduced as age progresses [13]. Although it is well-documented that the gut microbiome consistently evolves in response to age-related environmental changes, there remains a lack of knowledge on how this impacts the community’s response to microbial modifiers, such as prebiotics, probiotics, or other therapeutic administrations. Understanding the relationship between age, the gut microbiome, and its response to therapeutics becomes increasingly important when considering the application of precision medicine, which is personalized treatment tailored to an individual, because specific treatments may not remain effective, or produce desired effects to the same extent, as a person ages [14].

Exemplifying age-directed changes that occur to the gut microbiome is the genus *Bifidobacterium*, which is well-studied due to its known positive health benefits and is often used as a probiotic itself, or as the target of prebiotic administration [15,16]. This is a prominent taxa following birth, but upon reaching adulthood it is estimated that the gut microbiota contains only 0–18% *Bifidobacterium*, which is further reduced as aging progresses [17]. Additionally, the prominent species of *Bifidobacterium* present in the community is age-dependent; species *B. longum*, *B. infantis*, and *B. bifidum* that preferentially metabolize human milk oligosaccharides (HMOs) are more abundant in breastfed infants (BF infants) while *B. adolescentis*, which metabolizes other complex carbohydrates such as starch, is more abundant in adults [15,18]. Research has demonstrated that levels of *Bifidobacterium* within the gut microbiota positively corresponds with longevity and overall human health and negatively correlates with immunoscenescence, metabolic disorders, and other conditions such as IBS in elderly adults [17,19].

Although age is known to affect the abundance and dominant species of *Bifidobacterium* present in the community, there is minimal information available on how these age-related changes may influence the community’s response to a prebiotic with bifidogenic properties, such as 2’-fucosyllactose. 2’FL is a soluble milk glycan undigested in the upper gastrointestinal tract that is fermented by the gut microbiota producing positive benefits to both the microbial community and human health [20]. Both *in vitro* and *in vivo* studies have reported that 2’FL produces a strong bifidogenic effect in infants and toddlers [21,22]. Due to these reported health benefits, 2’FL has gained interest as a potential prebiotic for all age groups, expanding its application beyond early life up to end of life [23,24].

To provide critical information on the relationship between age and the gut microbiota, filling in these identified gaps in knowledge, a comparative evaluation was performed using the *ex vivo* Systemic Intestinal Fermentation Research (SIFR^®^) technology. Gut microbial communities were cultured from subjects spanning 0–70 years of age divided into six distinct age groups: breastfed (BF) infants (0–0.3 y), toddlers (1–1.5 y), children (5–7 y), and three groups of adults (25–35 y, 35–50 y, and 50–70 y) [25]. Data generated from metagenomics, flow cytometry, metabolic quantification, and REIMS analysis were combined in order to provide a comprehensive understanding of how age affects the gut microbiome in terms of structure, function, and phenotype. To further expand on this topic, the influence of age on the community’s response to therapeutic administration was tested using the well-known prebiotic 2’FL. The results of this study provided granularity on the changes to the gut microbiome that correspond with chronological age and how this may impact a therapeutic response. Of particular importance were the changes identified to species within *Bifidobacterium*, which were found to be highly age-dependent in terms of dominant species and community abundance. Together, the results demonstrate the importance of age when considering therapeutic administration and its role in the development of precision medicine.

## 2. Methods

Test products: Test products 2’FL, (Glycom, GlyCare^TM^ 2FL 9000, DSM-Firmenich, Maastricht, The Netherlands), Fructooligosaccharide (FOS) from chicory root, (F8052, Sigma-Aldrich, St. Gallen, Switzerland), and lactose, (6868, Carl Roth, Karlsruhe, Germany) were purchased commercially. All tested products were used at a concentration of 5 g/L. Additionally included was an unsupplemented parallel control (no substrate control = NSC), in which the fecal microbiome was grown in the absence of additional test products [25,26].

**Figure 1:**
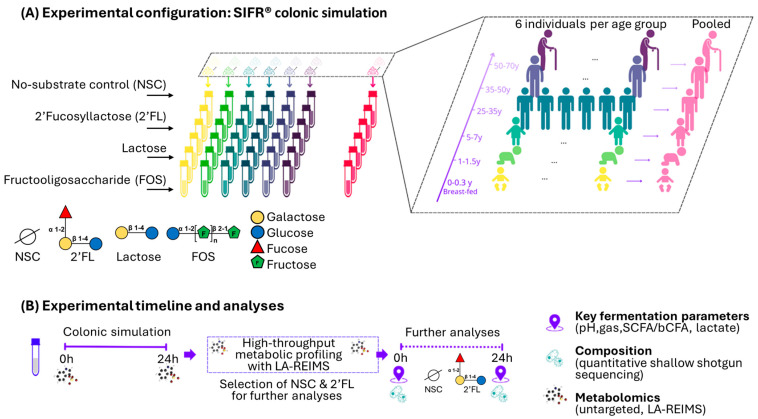
Schematic depicting the experimental design using the *Ex vivo* SIFR^®^ technology to examine the impact of 2’FL (along with FOS and lactose) on the gut microbiota of six different age groups, ranging from BF infants over toddlers and children up to three adult groups (25–35, 35–50, 50–70) compared to an unsupplemented parallel control (NSC). For each age group, incubations were conducted for six individual microbiomes, along with an incubation with a single pooled sample of the six microbiomes. SIFR = Systemic Intestinal Fermentation Research; 2’FL = 2’Fucosyllactose; FOS = fructo-oligosaccharides; NSC = no substrate control; SCFA = short-chain fatty acids; bCFA = branched chain fatty acids, LA = lactate; LA-REIMS = Laser-assisted rapid evaporative ionization mass spectrometry.

*Ex vivo* experimental design: Feces were harvested from subjects living in Belgium that fell within one of the six following age categories at a 1:1 male to female ratio: BF infants (0–0.3 y), toddlers (1–1.5 y), children (5–7 y), adults (25–35 y), adults (35–50 y), adults (50–70 y). All adults had a BMI < 30 and were non-smokers, consumed less than three alcoholic beverages a day, were antibiotic free, prebiotic free, and probiotic free for at least three months, not pregnant or lactating, had no GI disorders including cancers, ulcers, and inflammatory bowel disease (IBD), and were not taking any anti-psychotics or anti-allergy drugs (Table S1). Fresh feces were collected according to a procedure approved by the Ethics Committee of the University Hospital Ghent (reference number BC-09977) that required a signed informed consent. For underage participants, the responsible adult provided consent. *Ex vivo* SIFR^®^ experiments were performed as recently described using a bioreactor management device (Cryptobiotix, Ghent, Belgium) (Figure 1) [25]. Briefly, individual bioreactors containing 5 mLs of nutritional medium (M001, Cryptobiotix, Ghent, Belgium) were inoculated with freshly collected feces from a single donor. For pooled samples, an equal volume of each donor’s sample within each age group were combined and used as the inoculum. Various test compounds were added, and bioreactors were sealed individually in an anaerobic environment, then incubated for 24 h at 37 °C with continuous agitation (140 rpm) (MaxQ 6000, Thermo Scientific, Merelbeke, Belgium) [27,28]. In an initial preliminary experiment, the effects of three known products with prebiotic qualities, lactose, FOS, and 2’FL were screened for activity using rapid evaporation ionization mass spectrometry (REIMS) to identify which substrate produced the largest change in phenotype based on significant features identified (Figure S1). Administration with 2’FL produced the most changes, specifically to lipids and lipid-like molecules, and was therefore selected for continued use in the experiment. Based on these results, 2’FL was selected to determine the impact of age on prebiotic potential, which was evaluated using a combination of in-depth analyses as described below. For DNA sequencing, a 1 mL volume of culture was centrifuged at 5000× *g* for 10 min, and the pellet was used for DNA extraction. The supernatant was separated and used for metabolomics and SCFA analysis. For flow cytometry, culture was harvested and used directly.

DNA sequencing and analysis: DNA was extracted via the SPINeasy DNA Kit for Soil (MP Biomedicals, Eschwege, Germany), according to the manufacturer’s instructions. Subsequently, DNA libraries were prepared using the Nextera XT DNA Library Preparation Kit (Illumina, San Diego, CA, USA) and IDT Unique Dual Indexes with total DNA input of 1 ng [26]. Genomic DNA was fragmented using Illumina Nextera XT fragmentation enzyme, and indexes were added to each sample followed by 12 cycles of PCR to construct libraries [29]. DNA libraries were purified using AMpure magnetic Beads (Beckman Coulter, Brea, CA, USA), eluted in QIAGEN EB buffer, quantified using a Qubit 4 fluorometer and a Qubit dsDNA HS Assay Kit, and sequenced on an Illumina Nextseq 2000 platform 2 × 150 bp. Unassembled sequencing reads were converted to relative abundances (%) using the CosmosID-HUB Microbiome Platform (CosmosID Inc., Germantown, MD, USA; accessed on 16 February 2022). This platform was also used to calculate the Chao1, Shannon, and reciprocal Simpson diversity index.

Bioinformatical analysis and statistics: All univariate and multivariate analyses were performed using R (version 4.2.2; www.r-project.org; accessed on 13 May 2024). This software was used to make a series of violin plots and heat maps. While violin plots present the actual values, heat maps present log_2_-transformed fold changes for the different treatments compared to the parallel control arm (NSC). For the principal component (PCA) analysis, the FactoMineR package was used [30]. Regularized Canonical Correlation Analysis (rCCA) was executed using the mixOmics package with the shrinkage method for estimation of penalization parameters (version 6.20.3) [31]. Significance of supplementation effects compared to parallel un-supplemented controls were assessed via repeated measures ANOVA analyses, with *p*-value correction according to Benjamini–Hochberg [32]. For the analysis of microbial composition, three measures were taken. First, the aforementioned statistical analysis was performed on log_10_-transformed values. Second, a value of a given taxonomic group below the limit of detection (LOD) was considered equal to the overall LOD. Finally, a threshold was set to retain the 100 most abundant species in the analysis.

SCFA quantification and environmental parameters: SCFAs (acetate, propionate, butyrate, and valerate) and branched-chain fatty acids (bCFA; sum of isobutyrate, isocaproate, and isovalerate) were determined using a Trace 1300 chromato-graph (Thermo Fisher Scientific, Merelbeke, Belgium) equipped with a Stabilwax-DA capillary GC column, a flame ionization detector, and a split injector using nitrogen gas as the carrier, as previously described [25,29]. Environmental pH was measured using an electrode (Hannah Instruments Edge HI2002, Temse, Belgium) [25,26]. Gas was measured at the beginning and end of the experiment [29]. Lactate was quantified using an enzymatic method (EnzytecTM, R-Biopharm, Darmstadt, Germany). To determine total bacteria counts, samples were diluted in anaerobic phosphate-buffered saline (PBS), followed by cell staining with SYTO 16 at a final concentration of 1µM, and counted via a BD FACS Verse flow cytometer (BD, Erembodegem, Belgium) [25,29]. Data were analyzed using FlowJo, version 10.8.1. Quantitative insights were obtained by correcting proportions (%; 16S rRNA gene profiling) with total counts (cells/mL; flow cytometry), resulting in estimated cells/mL of different taxa.

Rapid evaporation ionization mass spectrometry (REIMS) methodology: The analytical platform used was a modification of that previously reported [33] with a carbon dioxide laser (A.R.C., Nürnberg, Germany) used for sample heating and evaporation and an XYZ gantry robot (igus, Northampton, UK) used for automated movement of the laser focusing lens piece. The CO_2_ focusing lens was moved to ≈2 mm above the sample pellet within the well of a 96-well plate (Greiner Bio-One, Frickenhausen, Germany). The pellet was produced through centrifugation of 200 µL of sample for 10 m at 4000× *g* in a refrigerated (4 °C) chamber and supernatant removed. Each plate was analyzed in a column-by-column pattern with sample heating occurring for approximately 5 s, followed by 10 s to allow washout of the evacuation tubing. The CO_2_ laser was set at 2 W and 20 Hz pulsatile operation. The analyte-containing smoke produced through sample heating was aspirated through PTFE tubing with a 1.5 mm internal diameter. This was linked to the CO_2_ focusing lens through a 3D resin-printed capture head that channels the Xevo G2-XS QToF (Waters, Wilmslow, UK) mass spectrometer’s native vacuum for smoke evacuation. The PTFE tubing was linked to a stainless-steel T-piece that allowed mixing of the analyte-containing smoke with 2-propanol solvent containing leucine enkephalin at a concentration of 0.1 ng/µL introduced at a flow rate of 250 µL/m using an Acquity I-Class Plus binary solvent manager (Waters, Milford, MA, USA). The combined mixture entered the REIMS interface (Waters, Wilmslow, UK), where it was heated to approximately 700 °C via collision with a Kanthal ribbon surface to remove the 2-propanol solvent prior to entry into the ion guide of the mass spectrometer. Mass spectra were acquired over a 50 to 1200 *m/z* range at a scan rate of two scans per second in negative ion detection mode.

REIMS Statistical Analysis: Acquired mass spectra were imported into Abstract Model Builder software (Version 1.0.1966.0, Waters, Budapest, Hungary), where it underwent lockmass correction, background subtraction, and peak binning to 0.1 Da-width bins. Data was exported as a comma-separated value file and processed within the R environment (Version 4.2.1) in R Studio (Version 2023.03.0+386). Any features which had a significant (*p* < 0.05) correlation value of more than 0.9 through Spearman correlation against the leucine enkephalin signal at bin 554.25 were removed from further analysis. MetaboAnalyst 5.0 [34] was used for univariate (ANOVA) and multivariate (PCA) analysis after data was subjected to total ion count normalization, Log_10_ transformation, and Pareto scaling. GraphPad Prism (Version 9.5.1.733) and Microsoft Excel 365 were used for creation of summary figures. Significant REIMS features identified through analysis were tentatively identified against the Human Metabolome Database (downloaded 25 April 2022) using an in-house R script with an accuracy threshold of less than 10 ppm, and with no preferential adduct selection. Correlation between OTU and REIMS features was conducted in the R environment, as above, using Spearman correlation with a significant threshold of less than 0.05, after Bonferroni correction, and R threshold of less than −0.5 or more than 0.5.

## 3. Results

### 3.1. Age Drives Divergence in Community Structure

**Figure 2:**
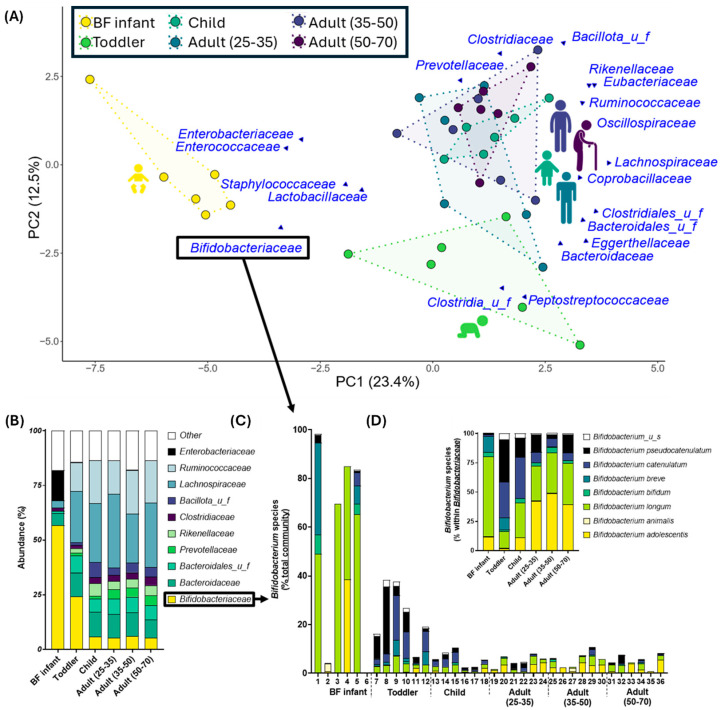
The gut microbiota was divergent across multiple age groups. (**A**) Principal Components Analysis (PCA) depicting the community structure for each test subject in relation to one another, with the 20 most dominant families being highlighted; (**B**) Abundance (%) of 10 most abundant families, averaged per age group (n = 6); (**C**) Absolute abundance of *Bifidobacterium* for each subject tested; (**D**) Relative abundance for each *Bifidobacterium* species (normalized compared to total *Bifidobacterium* levels), averaged per age group (n = 6).

Shotgun sequencing revealed that each subject used for this study produced a unique gut microbiome and that the communities clustered together according to age group (Figure 2A). The largest difference was observed between the BF infant age group and all others, represented by large separation along PC1 (23.4%). Both the BF infant and toddler groups were significantly distinct from all other age groups, whereas children and older were more similar in composition (Table S2). Nevertheless, LefSe analysis identified core taxa that were significantly enriched in each of the six age groups tested, indicating that a core microbiome could be identified for each age group (Table S3).

The strongest age-dependent difference in microbiota composition was the decrease in *Bifidobacteriaceae* as age increased (Figure 2B). In four of six BF infants, the microbiota was almost completely comprised of *Bifidobacterium* species (Figure 2C), but levels of *Bifidobacterium* decreased to < 40% of the community for subjects in the toddler group and were further reduced to < 10% for children and adults (Figure 2B). Not only did the prevalence of *Bifidobacterium* decrease with age, but the species that dominated the communities were highly age-dependent (Figure 2C,D). For the BF infant group, the most dominant species were *B. longum* followed by *B. breve* and *B. bifidum.* This changed markedly by 1–1.5 years of age; for toddlers and to lesser extent also for children, *B. catenulatum* and *B. pseudocatenulatum* were most prevalent. Finally, for all three adult groups *B. adolescentis* was the dominant *Bifidobacterium* species.

### 3.2. Age-Related Differences in Community Structure Drove the Response to 2’FL Supplementation, Exemplified by Age-Dependent Bifidogenic Effect

**Figure 3:**
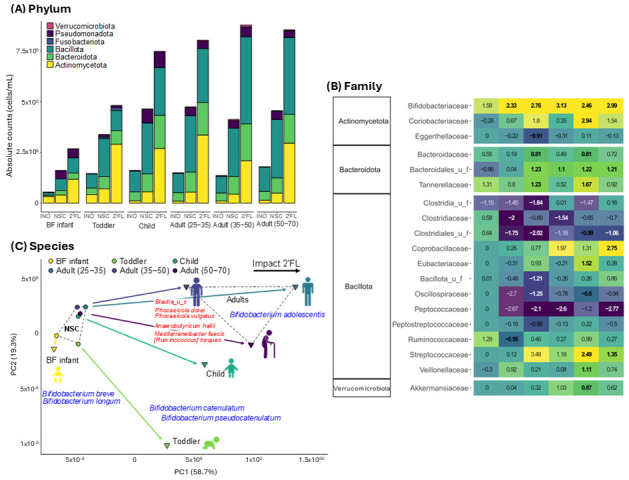
Treatment with 2’FL elicited structural changes to the gut microbiota, with an age-dependent effect on *Bifidobacterium* species. (**A**) Absolute levels at the phylum level for the original fecal sample at 0 h (INO) along with the untreated (NSC) and 2’FL treated communities at 24 h; (**B**) Heat map showing log_2_ fold change at the family level following 24 h of treatment with 2’FL. Significant differences are indicated by bold (FDR < 0.10); (**C**) PCA biplot based on centered values of microbial species (cells/mL). Bifidobacterium species are indicated in blue while other commensals are in red.

2’FL elicited changes to the gut microbiome structure, with a significant increase in levels of Actinomycetota (synonym Actinobacteria) for all age groups (Figure 3A). The corresponding increase in cell density for 2’FL-treated communities compared to the no substrate control (NSC) indicated its use as carbon source able to support cellular growth (Figure 3A). At the family level, the increase in Actinomycetota was shown to be due to an increase in *Bifidobacteriaceae* for all age groups. This achieved statistical significance for the toddler and older age groups and corresponded with a decrease in families such as *Peptostreptococcaceae*, Clostridiales_u_f, and to a lesser extent *Clostridiaceae*, and *Oscillospiraceae* (Figure 3B). At the species level, more detailed insights were obtained. First, the response to 2’FL treatment was largely age-dependent (Figure 3C). Interestingly, the divergence between the NSC and 2’FL communities was driven by age-dependent *Bifidobacterium* species: *B. breve/longum* mostly increased for BF infants (positioning downwards), *B. catenulatum/pseudocatenulatum* mostly increased for toddlers/children (positioning to right bottom), and *B. adolescentis* mostly increased for adults. These results demonstrated that 2’FL exerted bifidogenic effects that were dependent on subject age. In other words, age was a determining factor in which *Bifidobacterium* were present in the community and, thus, directly influenced which *Bifidobacterium* species responded to 2’FL administration. Beyond *Bifidobacterium*, analysis at the species level also indicated that several commensal species were enhanced by 2’FL, particularly for the adult microbiota including, amongst others, *Phocaeicola dorei*, *Phocaeicola vulgatus*, *Anaerobutyricum hallii,* and *Mediterraneibacter faecis*. The statistical significance of specific findings are provided in Figures S2 and S3.

### 3.3. Pooled Communities Only Provided Indicative Insights into the Response of the Gut Microbiota

**Figure 4:**
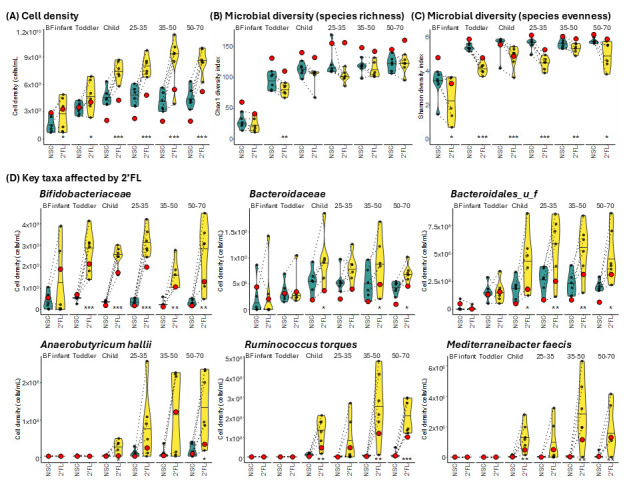
Pooled fecal communities provided indicative insights into the response of the gut microbiota to 2’FL treatment. (**A**) Cell density quantified by flow cytometry; Microbial diversity based on the (**B**) Chao1 diversity index; and (**C**) Shannon index; (**D**) Abundance of key microbial families and species affected by 2’FL treatment. Statistical significance was determined using repeated measures ANOVA with FDR correction. Adjusted *p*-values are indicated as follows: *p* < 0.1 *, *p* < 0.05 **, *p* < 0.01 ***. Pooled communities shown as a red dot. Headings 25–35, 35–50, and 50–70 are used to indicate the age groups Adults (25–35), Adults (35–50), and Adults (50–70), respectively.

An additional research question was to assess the effect of combining microbiomes from multiple subjects to a single pooled microbiome. After creating a pooled sample per age group, this was treated with 2’FL and the results were compared to the findings of the six individual microbiomes to determine whether or not they would produce similar results. In terms of bacterial load determined by flow cytometry, the pooled communities grew to a density similar to the averaged communities for the BF infants and toddler groups (Figure 4A). However, for the child and older groups, the pooled communities were substantially lower in density than the averaged communities.

In terms of microbial diversity, both species richness, determined by the Chao1 diversity index (Figure 4B), and community evenness, represented here using the Shannon’s diversity index (Figure 4C), were artificially high for the pooled samples. When looking at taxa that were most affected by 2’FL, the results of the pooled sample provided only indicative insights (Figure 4D). With some exceptions, the direction of the response for the pooled microbiome was generally in line with the response across the six individual donors. However, the pooled sample did not recapitulate the interpersonal differences in treatment response. For instance, for BF infants, only two out of six subjects responded with a bifidogenic effect to 2’FL treatment. Based on the result of the pooled sample, such insight in responders and non-responders was not provided as the pooled sample only revealed a marked bifidogenic effect for BF infants (likely due to the presence of 2’FL-fermenting *Bifidobacterium* species of the two responders). Overall, results with the pooled microbiota sample were thus rather indicative.

### 3.4. 2’FL Treatment Altered Metabolic Output and Community Phenotype in a Manner Irrespective of Age, Which Was Poorly Recapitulated by Pooled Communities

**Figure 5:**
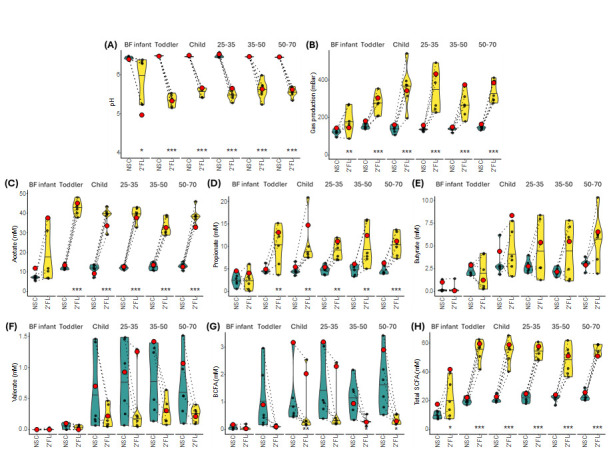
2’FL treatment altered metabolic output and community phenotype in a manner irrespective of age, which was poorly recapitulated by pooled communities. Metabolic output in terms of (**A**) environmental pH, (**B**) gas production, (**C**) acetate, (**D**) propionate, (**E**) butyrate, (**F**) valerate, (**G**) BCFAs, and (**H**) total SCFAs. Statistical significance was determined using repeated measures ANOVA with FDR correction. Adjusted *p*-values are indicated as follows: *p* < 0.1 *, *p* < 0.05 **, *p* < 0.01 ***. Pooled communities are shown as a red dot. Headings 25–35, 35–50, and 50–70 are used to indicate the age groups Adults (25–35), Adults (35–50), and Adults (50–70), respectively.

To assess the effect of age on functional changes driven by the addition of 2’FL, metabolic output for each community was assessed through monitoring of the fermentation parameters- environmental pH and gas production, and quantification of short chain fatty acid (SCFA) and lactate production (Figure 5). 2’FL markedly decreased pH, most likely due to the increase in total SCFAs, and increased gas production, which achieved statistical significance for all age groups (Figure 5A–C). For toddlers and older, elevated total SCFAs was driven by increased acetate production, which is a known metabolic byproduct of *Bifidobacterium*, and propionate (Figure 5D,E) [35,36]. There were no statistically significant changes observed in butyrate, valerate, or branched SCFAS (BCFA) for any age group (Figure 5F–H). For infants this was due to a lack of production, but for toddlers and older this was due to large variability between subjects. In all age groups, following both the NSC and 2’FL treatment, the pooled communities aligned closely with a single donor in each age group, suggesting that the pooled community adopted characteristics of a single donor and was not representative of the average response (Figure S4). Together, the results demonstrated that pooled communities may have been able to fairly represent a community under control conditions, but they were unable to accurately portray a pattern of response to a stimulus, shown here using 2’FL as an example, and could be described as metabolic outliers.

### 3.5. Associations Between Structure and Metabolic Output Were Highly Age-Dependent

**Figure 6:**
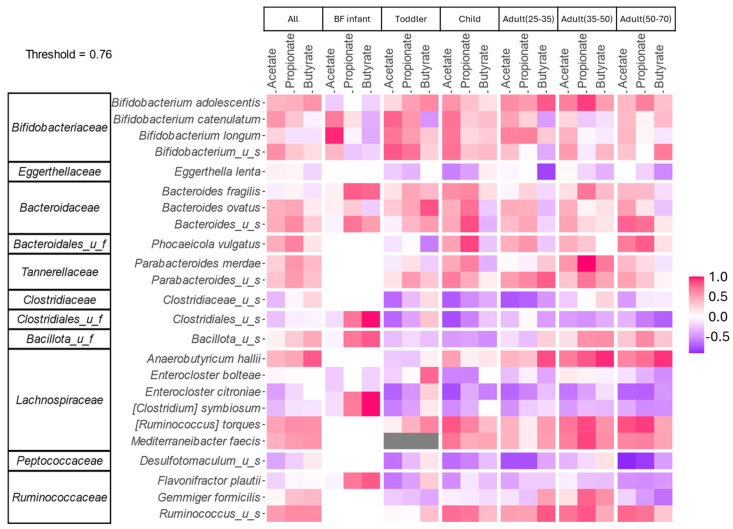
Correlation between structure and function for each age group. Taxa were limited to less than 20 for consideration. Only categories with a correlation > 0.5 or < −0.5 are depicted.

A correlation analysis was performed between metagenomic data and metabolic output in order to shed light on which taxa may be associated with production of the predominant SCFAs (Figure 6). Interestingly, for all age groups, *Bifidobacterium* species correlated positively to acetate production. Except for BF infants, *B. adolescentis* correlated positively with acetate, propionate, and butyrate, although to varying degrees. Looking at propionate and butyrate, which are not end products of 2’FL metabolism but were considered to have increased through cross-feeding interactions, the taxa correlated to these metabolites were highly age-dependent. Notably, for adults (25–35 y) and adults (35–50 y) there was a strong positive correlation between butyrate and *Anaerobutyricum hallii*. This correlation was also observed for adults (50–70 y), but to a lesser extent. This supported previous findings that metabolic byproducts released by *Bifidobacterium* in response to 2’FL administration are used by *A. hallii* to produce butyrate [37]. This observation was limited to the more mature microbial communities, as the child group had the same trend of increase for both of these taxa as well, but to a lesser extent. This indicated that age-specific cross feeding reactions were occurring.

### 3.6. Age-Dependent Structure-Function Correlations Were Detected Using REIMS Regardless of 2’FL Administration

**Figure 7:**
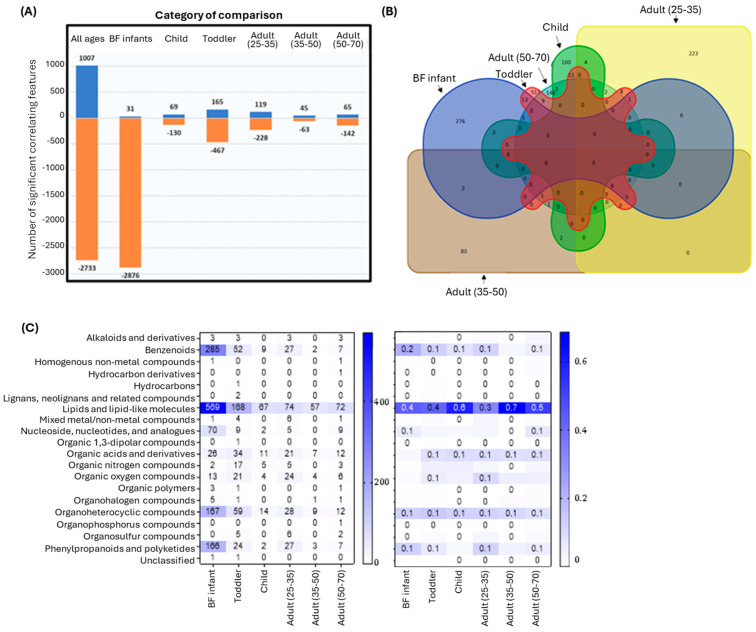
Spearman’s correlations between taxonomic structure and REIMS features. Significant correlations were considered as *p* < 0.05 after Bonferroni correction and R value above 0.5 or below −0.5. (**A**) Bar plot depicting significant correlations observed with positive correlations in blue and negative correlations in orange; (**B**) Venn diagram depicting the number of unique correlations between OTU and REIMS features; (**C**) Heat maps showing chemical taxonomies of the different correlations at the superclass level. Left: absolute numbers. Right: proportional within each experimental class.

To delineate the structure-function relationship for communities of different age groups, a Spearman’s correlation was conducted between taxonomic data and REIMS features. For this analysis, NSC and 2’FL treated communities within each age group were combined to increase statistical power. Both positive and negative correlations were identified, but there were more negative correlations observed for all age groups, with the largest number found within the BF infant group and smallest within the Adults (35–50 y) group (Figure 7A). The number of correlations unique to a specific age group were calculated and displayed as a Venn diagram, which highlighted that there were a number of significant correlations that were age-dependent, and a number of correlations that were shared (Figure 7B). To provide further resolution, the chemical taxonomies for each correlating feature specific to an age group were identified at the superclass level and portrayed as heat maps presenting the absolute values and ratios (Figure 7C). Based on this data, lipids and lipid-like molecules were by far the most prevalent feature for all age groups, most likely due to their increase following 2’FL administration (Figure S1), but other chemical super classes that contributed to age-distinction were benzenoids, organic acid derivatives, organic oxygen compounds, organohetercyclic compounds, and phenylpropanoids and polyketides. Together, this data demonstrated that the communities developed phenotypes unique to their respective age groups.

## 4. Discussion

It is well-recognized that the gut microbiome evolves in concert with its host over a lifetime, driven by changing conditions that alter the intestinal environment, i.e., diet, and/or epigenetic changes that occur due to cellular senescence [7,38,39,40]. Yet reports addressing age and the gut microbiota tend to highlight either early childhood, infants and BF infants, or elderly adults developing frailty. To provide much-needed clarification on how the gut microbiota is altered due to the aging process, in this study, comparative evaluation combining both metagenomics and metabolomics was performed on communities cultured from subjects spanning 0–70 years of age, divided into six distinct age groups, BF infants (0–0.3 y), toddlers (1–1.5 y), children (5–7 y), and adults belonging to three age ranges (25–35 y, 35–50 y, and 50–70 y). These communities were then treated with 2’FL to determine how the age-related changes to the gut microbiota translated to its ability to respond to therapeutic treatment. This was accomplished using an *Ex vivo* strategy (SIFR^®^ technology), which has been previously validated and found to provide results that are predictive of clinical outcomes [24,25].

When looking simply at community structure, the largest shift related to age was observed between BF infants and all others, which was driven by the reduction in *Bifidobacteriaceae* corresponding with an increase in taxa associated with the mature gut microbiota, i.e., members of Bacillota and Bacteroidota (Figure 1A) [41]. Although distinct from the BF infant group, the toddler group remained mostly separate from the other groups, whereas the child and older age groups were structurally similar and more closely clustered together. These observations confirmed previous reports that the gut microbiota reaches maturity following the first few years of life and, therefore, the toddler group (1–1.5 y) represented a transitory community between infancy and maturity [9,10,40]. Once the mature community was established, here this refers to the child group (5–7 y), age-driven shifts were comparably more minimal. This pattern of progression was also observed when looking at community phenotype, profiled by combining metagenomic and REIMS data to identify correlating features between the community structure and function (Figure 6). In this analysis, the toddler group had the most age-specific correlations and the most shared correlations, pointing towards the toddler age group as transitionary between the BF infant and mature communities (child and older age groups). Although structurally the differences between the child and older groups were less apparent, phenotypically there were a number of structure-function correlations that changed with age even once the community was mature, providing further evidence towards the development of specific age-related features.

Exemplifying the changes to the gut microbiota that correspond with age, there was an identified successive switch in prevalent species of *Bifidobacterium* and their overall abundance in the communities, similar to what has been reported previously [42,43]. Here, the transition from BF infants to the child and older groups was marked by a stepped decrease in the abundance of *Bifidobacterium*, which was considered most likely due to the introduction of foods during weaning [40,44]. Levels of *Bifidobacterium* were further reduced between the toddler group and older groups, with the dominant species of *Bifidobacterium* converting from those able to utilize human milk components (*B. longum*, *B. bifidum*) in the BF infants to those preferentially metabolizing complex carbohydrates (*B. adolescentis*) in the adult groups [15,18]. It was considered likely that the changes to the gut microbiota structure corresponding with age were directly related to diet, as the types and amount of food consumed changes with age and is known to play a major role in shaping the gut microbiota [13,38]. The observed switch in *Bifidobacterium* supported this supposition, although it is not possible to know how much of an influence diet has on age-related changes to the gut microbiome from this data set.

The divergence in community structure between age groups was further realized upon treatment with the prebiotic 2’FL, an HMO with known prebiotic properties and a well-established bifidogenic effect [22,45,46]. Here, 2’FL was administered to provoke a measurable reaction that was used to identify how the age-related changes to the gut microbiome influenced the response of the communities to a stimulus. Here, the addition of 2’FL statistically increased levels of *Bifidobacteriaceae* for all age groups, except for the BF infants (Figure 2B,C). It was hypothesized that the increase in *Bifidobacteriaceae* for the infant group failed to reach significance due to large interpersonal differences that obscured the statistical results and because the communities within this age group were already predominantly *Bifidobacterium* (four of six subjects), and these subjects were already consuming HMOs in their diet.

Notably, the results here found that the species of *Bifidobacterium* that responded to 2’FL was highly age dependent and seemed to be a primary factor driving divergence of the age-specific communities. It was considered that this observation was most likely due to the changes in the species of *Bifidobacterium* present, and their abundance, that occurred with age, and not necessarily due to selection by 2’FL. For BF infants, *B. breve* was the most affected, which was logical as *B. breve* is typically associated with the infant gut and not the adult gut [42]. For toddlers and older, the species of *Bifidobacterium* that responded to the highest degrees were *B. catenulatum*, *pseudocatenulatum*, and *adolescentis*, although only the increase in *B*. *adolescentis* reached statistical significance for all of these age groups. It is impossible to know from this study whether or not these species were enhanced due to their direct metabolism of 2’FL or through cross-feeding interactions. There are strains of *B. pseudocatenulatum* and *catenulatum* able to metabolize fucosyllated HMOs to varying degrees [47,48,49,50]; yet *B. adolescentis* is primarily associated with metabolism of plant-based carbohydrates and considered unlikely to metabolize HMOs or other human-derived glycans, indicating that the increase to this taxon may be due to cross-feeding or other interactions [51,52].

Although the largest effect on community structure was the increase in *Bifidobacteriaceae*, treatment with 2’FL produced a number of other taxonomic shifts that drove the age-specific communities further away from one another when visualized by PCA (Figure 2B). It was considered that, similar to *B. adolescentis*, these were likely due to cross-feeding interactions as there have been previous reports of cross-feeding between *Bifidobacterium* and other butyrate producers such as *A. hallii* [19,53,54,55,56]. Expanding on this further, in this study, the taxa that were enhanced due to potential cross-feeding were age-dependent, a unique and important finding. This was apparent in the heat map showing the taxonomic changes that occurred following the addition of 2’FL (Figure 3B) but was also confirmed using correlative analysis (Figure 5A). Highlighting this finding was the identified correlation between *B. adolescentis*, *A. hallii*, and butyrate. This correlative network is similar to what has been previously proposed, that metabolic byproducts released by *Bifidobacterium* (for example, lactate) are used by *A. hallii* with the end production of butyrate, either directly or through a more complex interaction [55,57]. This correlation was only found in the adult age groups even though both *B. adolescentis* and *A. hallii* were also present in the child group, albeit at a lower abundance than the adults. Yet for the child group *B. adolescentis* and *A. hallii* were not correlated to butyrate levels. In this case, here, the identified correlation between *B. adolescentis*, *A. hallii*, and butyrate was age specific. This is quite important when considering a prebiotic treatment as it is difficult to elucidate exactly how the prebiotic will be metabolized and how this will influence the resident members of the community. Where administration of a prebiotic to selectively enhance levels of a specific taxa, or subset of taxa, could be categorized as a targeted therapeutic approach, the cross-feeding reactions that occur as a byproduct are not targeted and can be highly variable depending on community composition, and here this was shown to be dependent on age.

In terms of metabolic output, the observed increase in SCFAs following 2’FL administration in this study was expected and aligns with results from previously published reports [56,58]. The only difference in SCFA release observed here was between the BF infants, which did not have any statistical differences in SCFAs following 2’FL treatment, and all other communities, which had a large increase in levels of total SCFAs following 2’FL treatment (Figure 4B). In fact, it was notable that the toddler and older age groups produced very similar patterns of SCFA release under NSC conditions and responded to 2’FL treatment with a strong increase in acetate, which indicated that this response was not affected by the age of the subjects. In this case, the differences observed in community structure did not affect SCFA production. The increase in acetate was considered most likely due to the metabolism of 2’FL by *Bifidobacterium* itself, which releases acetate and lactate following metabolism of hexoses via the bifid shunt [59,60]. On the other hand, changes to levels of propionate and butyrate in response to 2’FL were highly variable between subjects. Since propionate and butyrate are not identified end products of 2’FL metabolism, it was assumed that any changes to their concentration were due to cross-feeding between community members, which was discussed in the paragraph above.

To provide a more dimensional view of metabolic function, SCFA quantification was complemented with REIMS analysis, which provided data on the full complement of metabolites released. When looking at the metabolic profiles determined by REIMS (Figure 4C), age-specific patterns were detectable when visualized by PCA under NSC conditions. This indicated that while the pattern of SCFA release was similar for toddlers and older, there were other metabolites produced that drove age-specific separation between these communities. Yet the addition of 2’FL caused these metabolic profiles to converge, with less distinct separation between age groups. This indicated that the metabolites produced by 2’FL metabolism were not all necessarily age-dependent. Together these results indicated that subject age affected the overall structure of the gut microbiota, which in turn affected which taxa were enhanced due to 2’FL administration, yet the metabolic fate of 2’FL itself was similar across the multiple age groups. This was considered logical as the metabolic breakdown of 2’FL would not necessarily differ among communities, but the taxa that perform the metabolism and those affected by the released byproducts would be dependent on community composition.

Although not the primary subject of this study, the relevance of data generated from pooling gut microbiome communities was evaluated as this is a common strategy employed for microbiome testing [61,62]. Pooling, or combining samples from multiple donors together to create a single inoculum (N = 1), is beneficial as it increases diversity of microbes tested without having to increase the number of individual replicates required. Contrary to previous findings that pooled communities produced results similar to those of single donors [63], the results here found that pooled communities did not effectively recapitulate the average response (Figure 3 and Figure 4). While pooling did enrich the communities, which aligned with previous findings, here, pooling altered the structural changes that occurred due to 2’FL administration [63]. Furthermore, without the interindividual variation that occurs when comparing the response of the communities to treatment, statistical analysis was limited. Metabolically, the pooled communities seemed to take on the characteristics of a single subject (Figure 4), which was most apparent in the metabolic profiles determined using REIMS, where the pooled communities converged together following 2’FL treatment (Figure 4C). Together, this leads to the conclusion that pooled communities were not able to provide valuable information on the response to stimuli that may be variable among subjects.

## 5. Conclusions

In conclusion, the metagenomic results here indicated that subject age affected the community structure of the microbiome, and this translated into an age-dependent response of the communities to 2’FL treatment. In other words, the age of the subject was a driving factor in the community’s response to 2’FL in terms of structure and which taxa were affected through cross-feeding interactions. In terms of community phenotype of metabolic profiles, there were age-specific distinctions that were detected. However, the metabolic fate of 2’FL was similar between the toddler and older age groups. The results confirmed age as a key determinant of the gut microbiome, which is an important finding because it demonstrates that age should be considered when studying therapeutic treatments, such as prebiotics/probiotics, or functional foods, or when considering an approach to modify the gut microbiome using precision medicine.

## Data Availability

Data has been deposited in the NCBI SRA archives with Bioproject number PRJNA1177813.

## Supplementary Materials

The following supporting information can be downloaded at: https://www.mdpi.com/article/10.3390/nu17010151/s1, Table S1: Subject information; Figure S1: Selection of 2’FL; Table S2: Beta-diversity analysis indices: Table S3: Lefse analysis: Figure S2: Heatmap depicting structural changes directed by 2’FL; Figure S3: Age-dependent changes to *Bifidobacterium*; Figure S4: Functional PCA analysis.
