# Supplementary material for: The Bifidogenic Effect of 2’Fucosyllactose Is Driven by Age-Specific Bifidobacterium Species, Demonstrating Age as an Important Factor for Gut Microbiome Targeted Precision Medicine
Source: Nutrients. 2024 Dec 31;17(1):151. doi: 10.3390/nu17010151 (PMC11723031; doi:10.3390/nu17010151)
Supplement: Supplementary file 1 [file nutrients-17-00151-s001.zip › nutrients-3392092-supplementary.pdf]

Table S1. Information (sex and age) of each subject included in the study, along with characteristics of the fecal donation they provided (Bristol stool score (BSS) and pH). The test subjects belonged to six different age groups (breastfed (BF) infants, toddler, kid and adults (25–35, 35–50, and 50–70 years of age)).

Donor	Age group	Sex	Age (y.m)	BSS	pH
1	BF infant	Female	0.3	6	4.64
2	BF infant	Female	0.1	6	6.64
3	BF infant	Female	0.0	6	5.2
4	BF infant	Male	0.3	6	6.95
5	BF infant	Male	0.3	6	5.07
6	BF infant	Male	0.4	6	6.49
7	Toddler	Female	1.5	5	5.99
8	Toddler	Female	1.5	3	7.16
9	Toddler	Female	1.0	6	5.59
10	Toddler	Male	1.5	5	6.84
11	Toddler	Male	1.0	4	7.37
12	Toddler	Male	1.5	4	721
13	Kid	Female	6	4	6.2
14	Kid	Female	6	4	7.14
15	Kid	Female	6	4	6.52
16	Kid	Male	6	4	6.08
17	Kid	Male	6	3	8
18	Kid	Male	9	3	6.62
19	Adult (25-35)	Female	27	4	5.86
20	Adult (25-35)	Female	31	4	7.19
21	Adult (25-35)	Female	34	4	6.91
22	Adult (25-35)	Male	31	4	6.71
23	Adult (25-35)	Male	28	4	6.61
24	Adult (25-35)	Male	31	5	5.48
25	Adult (35-50)	Female	39	3	6.65
26	Adult (35-50)	Female	41	4	6.92
27	Adult (35-50)	Female	42	3	7.07
28	Adult (35-50)	Male	37	4	5.95
29	Adult (35-50)	Male	40	4	6.7
30	Adult (35-50)	Male	50	4	7.24
31	Adult (50-70)	Female	65	4	7.13
32	Adult (50-70)	Female	61	4	6.89
33	Adult (50-70)	Female	58	4	7.08
34	Adult (50-70)	Male	67	3	6.4
35	Adult (50-70)	Male	58	5	6.77
36	Adult (50-70)	Male	61	4	7.06
37	Pooled samples from BF infants				
38	Pooled samples from toddlers				
39	Pooled samples from kids				
40	Pooled samples from adults (25-35)				
41	Pooled samples from adults (35-50)				
42	Pooled samples from adults (50-70)				

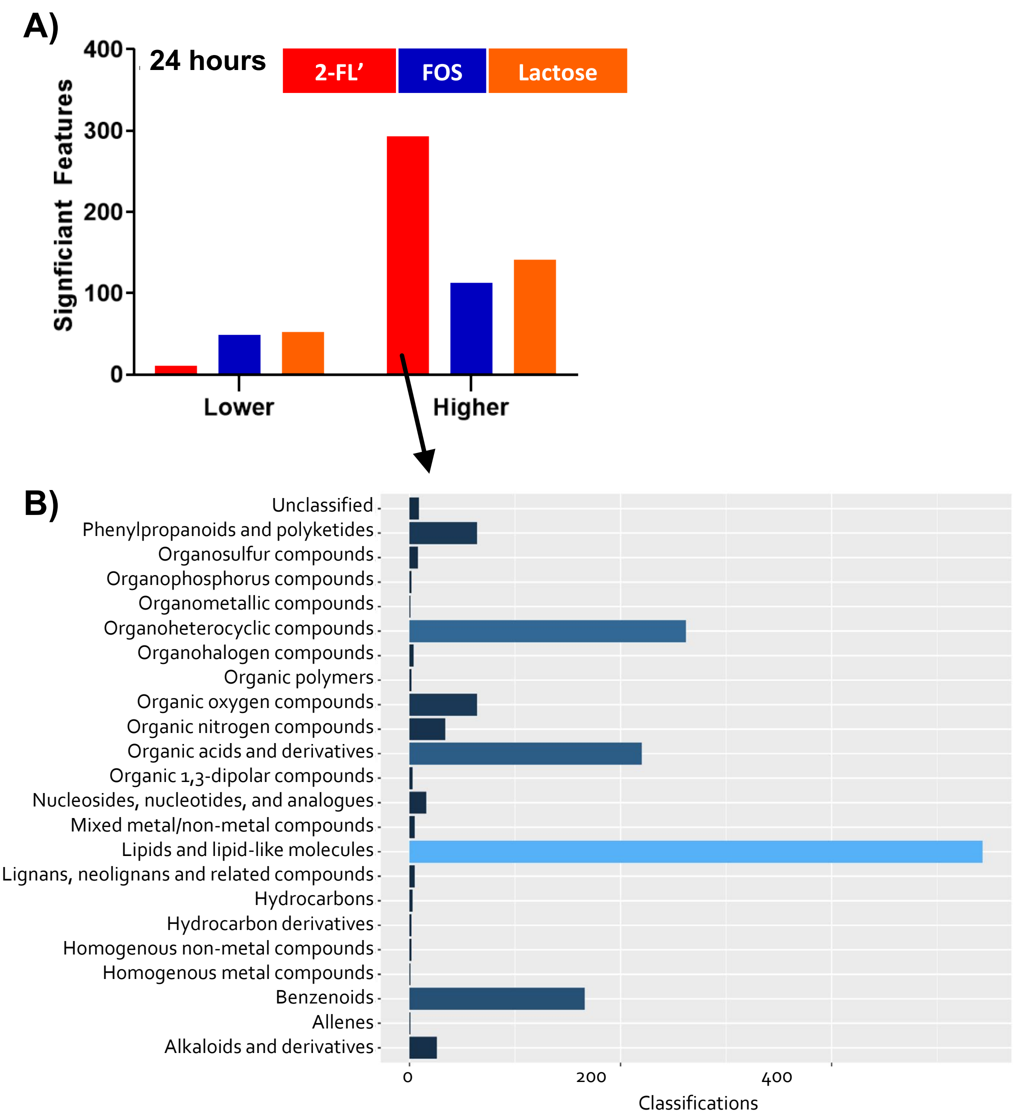

Figure S1. Administration with 2′FL produced the most changes, specifically to lipids and lipid-like molecules, and was therefore selected for in-depth analysis. (**A**) Number of features (as analyzed via REIMS) that were significantly affected (FDR = 0.05) by 2′Fucosyllactose (2-FL′), fructooligosaccharides (FOS), or lactose compared to an untreated study arm (NSC) at 24 h of growth. (**B**) The significant univariate features from the 2′Fucosyllactose group were tentatively identified against the Human Metabolome Database with an accuracy threshold of < 10 ppm. Classifications are given at the super-class level.

Table S2. Beta-diversity analysis via (A) Bray–Curtis and (B) Jaccard indices supported that the exploratory findings on differences in microbiome composition between age groups (shown in Figure 2) are significant.

A) **Bray-Curtis**

Cohorts	Sample Size	Permutations	Statistic	P-value
All	36	999	4.667	0.001
BF Infant ↔ Toddler	12	999	5.940	0.001
BF Infant ↔ Child	12	999	7.181	0.005
BF Infant ↔ Adult (25-35)	12	999	7.167	0.005
BF Infant ↔ Adult (35-50)	12	999	7.369	0.004
BF Infant ↔ Adult (50-70)	12	999	8.178	0.002
Toddler ↔ Child	12	999	3.479	0.004
Toddler ↔ Adult (25-35)	12	999	2.989	0.010
Toddler ↔ Adult (35-50)	12	999	4.200	0.002
Toddler ↔ Adult (50-70)	12	999	4.195	0.005

B) **Jaccard**

Cohorts	Sample Size	Permutations	Statistic	P-value
All	36	999	3.293	0.001
BF Infant ↔ Toddler	12	999	3.665	0.004
BF Infant ↔ Child	12	999	5.021	0.003
BF Infant ↔ Adult (25-35)	12	999	4.522	0.001
BF Infant ↔ Adult (35-50)	12	999	4.873	0.004
BF Infant ↔ Adult (50-70)	12	999	4.945	0.005
Toddler ↔ Child	12	999	2.821	0.003
Toddler ↔ Adult (25-35)	12	999	2.340	0.003
Toddler ↔ Adult (35-50)	12	999	3.333	0.003
Toddler ↔ Adult (50-70)	12	999	3.660	0.004

Table S3. Significant enrichment of species within each of the six groups (LefSe analysis; $p < 0.01$ to retain 60 species). Results are shown in the following format: LDA value/p-value.

Enriched for	Species	BF Infant	Toddler	Child	Adult (25-35)	Adult (35-50)	Adult (50-70)
BF Infant	*Enterococcus faecalis*	3.5/0.0072	-	-	-	-	-
	[Ruminococcus] gnavus	-	3.7/0.0022	-	-	-	-
	Anaerostipes hadrus	-	3.8/0.0011	-	-	-	-
	Bacteroides_u_s	-	4.5/0.0072	-	-	-	-
	Bifidobacterium catenulatum	-	4.6/0.0071	-	-	-	-
	Bifidobacterium_u_s	-	4/0.0027	-	-	-	-
Toddler	*Blautia wexlerae*	-	3.8/0.0038	-	-	-	-
	Clostridioides difficile	-	3.5/0.0025	-	-	-	-
	Eggerthella lenta	-	3.4/0.0013	-	-	-	-
	Eggerthella_u_s	-	3.5/0.0006	-	-	-	-
	Erysipelotrichaceae_u_s	-	3.1/0.0043	-	-	-	-
	Lachnospiraceae_u_s	-	4.7/0.0013	-	-	-	-
	Ruminococcus_u_s	-	4.3/0.0073	-	-	-	-
	Alistipes communis	-	-	3.2/0.0045	-	-	-
	Alistipes finegoldii	-	-	3.6/0.0075	-	-	-
	Alistipes_u_s	-	-	3.9/0.0039	-	-	-
	Bacteroides thetaiotaomicron	-	-	3.4/0.002	-	-	-
	Eubacterium ramulus	-	-	3.7/0.0014	-	-	-
Child	*Firmicutes_u_s*	-	-	4.6/0.0002	-	-	-
	Mediterraneibacter faecis	-	-	3.5/0.0006	-	-	-
	Odoribacter splanchnicus	-	-	3.1/0.0029	-	-	-
	Oliverpabstia intestinalis	-	-	3.6/0.0042	-	-	-
	Oscillibacter_u_s	-	-	3.9/0.0011	-	-	-
	Parabacteroides_u_s	-	-	4/0.0033	-	-	-
	Waltera intestinalis	-	-	3.8/0.0026	-	-	-
	Bacteroides ovatus	-	-	-	3.4/0.009	-	-
	Blautia massiliensis	-	-	-	3.8/0.0025	-	-
	Blautia_u_s	-	-	-	4.4/0.0004	-	-
Adult (25-35)	*Roseburia faecis*	-	-	-	4.2/0.0038	-	-
	Roseburia hominis	-	-	-	3.3/0.0029	-	-
	Roseburia intestinalis	-	-	-	3.8/0.008	-	-
	Roseburia_u_s	-	-	-	4.1/0.0018	-	-
	Adlercreutzia equolifaciens	-	-	-	-	3.4/0.0024	-
	Anaerobutyricum hallii	-	-	-	-	3.6/0.0016	-
	Barnesiella intestinihominis	-	-	-	-	3.2/0.0087	-
	Bilophila wadsworthia	-	-	-	-	3.1/0.0021	-
	Blautia obeum	-	-	-	-	3.7/0.0003	-
	Butyricicoccus_u_s	-	-	-	-	3.3/0.003	-
	Dorea formicigenerans	-	-	-	-	3.4/0.0067	-
Adult (35-50)	*Dorea_u_s*	-	-	-	-	3.3/0.0088	-
	Eubacterium ventriosum	-	-	-	-	3.2/0.0006	-
	Faecalibacterium prausnitzii	-	-	-	-	4.3/0.0081	-
	Gemmiger formicilis	-	-	-	-	4.1/0.0095	-
	Methanobrevibacter smithii	-	-	-	-	3.3/0.002	-
	Parabacteroides merdae	-	-	-	-	3.8/0.0031	-
	Romboutsia timonensis	-	-	-	-	2.7/0.0094	-
	Subdoligranulum_u_s	-	-	-	-	4.1/0.0002	-
	[Ruminococcus] torques	-	-	-	-	-	3.7/0.0001
	Blautia luti	-	-	-	-	-	3.3/0.0001
	Clostridium phoceensis	-	-	-	-	-	3.2/0.0008
	Clostridium_u_s	-	-	-	-	-	4.1/0.0035
	Collinsella bouchesdurhonensis	-	-	-	-	-	2.6/0.0072
	Coprococcus catus	-	-	-	-	-	3.2/0.0022
Adult (50-70)	*Desulfovibrio piger*	-	-	-	-	-	3.2/0.0014
	Dorea longicatena	-	-	-	-	-	3.9/0.0018
	Faecalibacterium_u_s	-	-	-	-	-	4.2/0.001
	Lawsonibacter asaccharolyticus	-	-	-	-	-	3.3/0.001
	Roseburia inulinivorans	-	-	-	-	-	3.4/0.0006
	Ruminococcaceae_u_s	-	-	-	-	-	4.4/0.0001
	Senegalimassilia anaerobia	-	-	-	-	-	3.2/0.0026

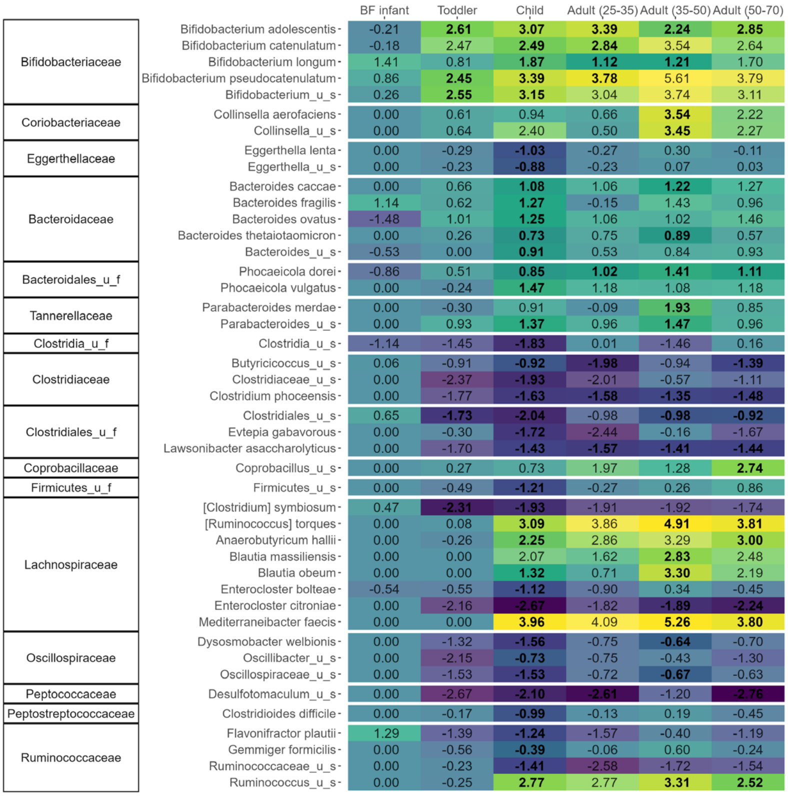

Figure S2. Treatment with 2′FL elicited age-dependent structural changes to the gut microbiota. Heat map showing $\log_2$ fold change at the species level following 24 h of treatment with 2′FL. Significant differences are indicated by bold (FDR < 0.10).

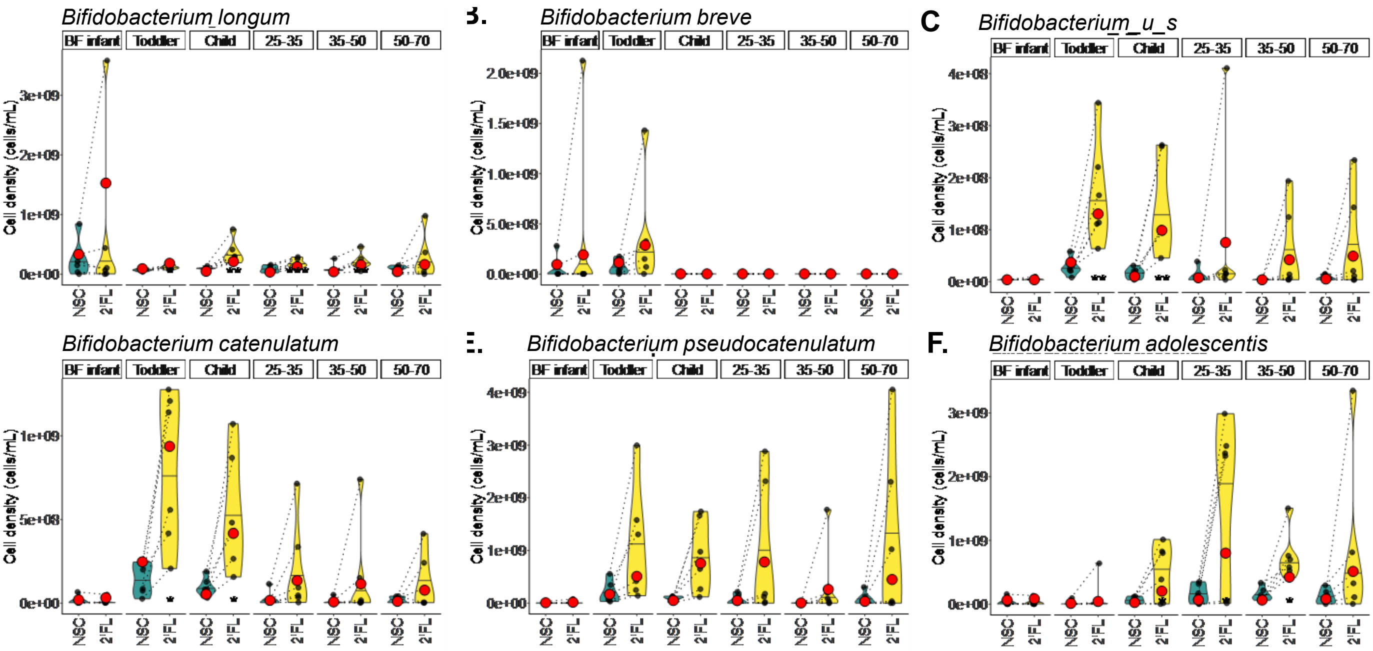

Figure S3. Treatment with 2′FL elicited age-dependent effect on *Bifidobacterium* species. Abundance of *Bifidobacterium* species affected by 2′FL treatment. Statistical significance was determined using repeated measures ANOVA with FDR correction. Adjusted *p*-values are indicated as follows: $p < 0.1$ *, $p < 0.05$ **, $p < 0.01$ ***. Pooled communities shown as a red dot.

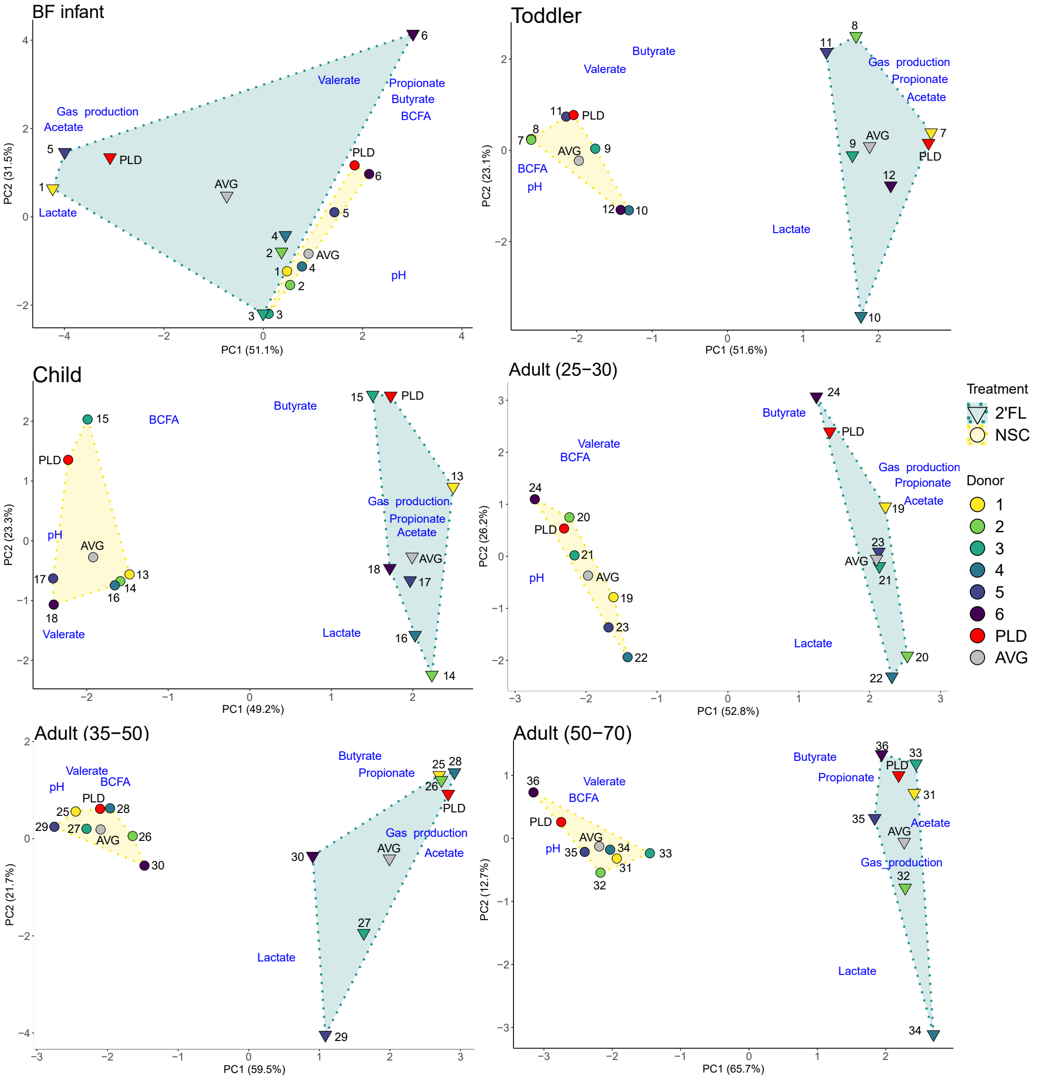

Figure S4. Metabolic output of communities in the NSC and 2′FL treatment conditions. PCA depicting shifts in community function following 2′FL treatment.
